# Symmetry breaking in thermal photonics

**DOI:** 10.1038/s41377-022-01044-8

**Published:** 2022-12-07

**Authors:** Xueji Wang, Zubin Jacob

**Affiliations:** Birck Nanotechnology Center, Elmore Family School of Electrical and Computer Engineering, Purdue University, West Lafayette, IN 47907 USA

**Keywords:** Optical materials and structures, Optical physics

## Abstract

Engineering symmetries in nanostructures and metasurfaces provides a new paradigm to control incoherent heat radiation for applications in energy conversion, thermal sources, infrared imaging, and radiative cooling.

Thermal radiation is omnipresent and is engineered for various applications in modern photonics, such as cooling, imaging, and energy harvesting. This has motivated a continued search for platforms to tailor the spectrum, polarization, and directivity of heat radiation. Unlike laser light which has been engineered extensively through various devices, heat radiation is incoherent and poses unique challenges. Recent efforts in the field of thermal photonics have achieved significant advances using infrared metamaterials but the ability to control thermal radiation is still limited by two key factors. First, photonic strategies generally act over a narrow spectral region, whereas thermal radiation has a broad spectrum that changes with temperature. Second, the material database of thermal photonics is limited as many materials drastically lose their stability at elevated temperatures.

Symmetries and symmetry breaking reveal a new pathway to control radiative heat flow and address existing challenges in the field. Symmetry describes the property of a physical system that remains invariant under certain transformations. In fundamental physics, symmetries and invariances have been proven to dictate the structures of the laws of nature^1^. In technological applications, a versatile design principle to obtain desired physical properties is emerging through the manipulation of symmetries. Symmetry breaking in thermal photonics gives a perfect illustration of such a technical route (Fig. 1). While thermal radiation microscopically originates from incoherent (random) thermal fluctuations, symmetries impose fundamental constraints that shape the macroscopic radiative behavior. For instance, time-reversal symmetry leads to the stringent equivalence between absorption and emission even for arbitrary complex media. Other symmetries related to spatial transformations can be exploited to engineer spin angular momentum buried inside the completely incoherent heat signal. Therefore, breaking symmetries through nanostructured media, nonreciprocal media and metasurfaces is heralding a new era of radiative thermal engineering.

Now, writing in this issue of *eLight*, Liu et al. provide a systematic overview of symmetry-based thermal radiation engineering^2^. They discuss the profound influence of symmetry-breaking on thermal radiation in the context of various novel physical phenomena. A variety of broken symmetries are aptly categorized into three groups: broken geometric symmetries, engineered mode symmetries, and broken reciprocity.

The authors first introduce the physical consequences of optical anisotropy and aperiodicity on thermal radiation. Strong anisotropy can arise in simple photonic structures such as gratings and multilayered structures even without intensive geometric optimization. This insight eases the construction of high-temperature-stable systems and promotes anisotropy-based strategies for extreme-temperature applications^3^. Anisotropic thermal antennas have a superior performance in tailoring the directionality of thermal radiation. Breaking of rotational symmetry is discussed as a design principle for controlling the angular far field pattern of thermal radiation. Opportunities exist for transitioning to industry applications since disordered aperiodic structures permit scalable manufacturing^4^.

Chirality is another important aspect of geometric symmetry which is tied to mirror- and inversion- symmetry breaking. Chiral thermal emission is a unique phenomenon that has been observed in condensed stars. Generating such chiral thermal emission in a symmetry-broken system is of both fundamental and applied interest. It can enable a compact circular-polarized light source in the mid-infrared spectral range that is desired in numerous scientific and industrial applications^5^. While laser light has been used to study chirality, spin angular momentum and orbital angular momentum of coherent radiation extensively, exploiting thermal agitation in metasurfaces to shape such properties in completely incoherent light is an unexplored frontier.

Fano resonance is a manifestation of the broken mode symmetry, which was first elaborated by Ugo Fano in 1961^6^. In thermal photonics, the asymmetric line shape in Fano resonance supplies a sharp transition from highly emissive to highly reflective (or transmissive) states. It offers an exceptional opportunity to enhance or suppress thermal radiation and control near-field heat transfer. Additionally, bound states in the continuum (BIC) is a unique phenomenon related to Fano resonance. Over the last few years, the topological interpretation of BICs has been under active investigation^7^. Exploration of BICs in this context may ultimately merge the fields of thermal photonics with topological physics, providing opportunities for topologically engineered radiative heat transfer.

**Fig. 1:**
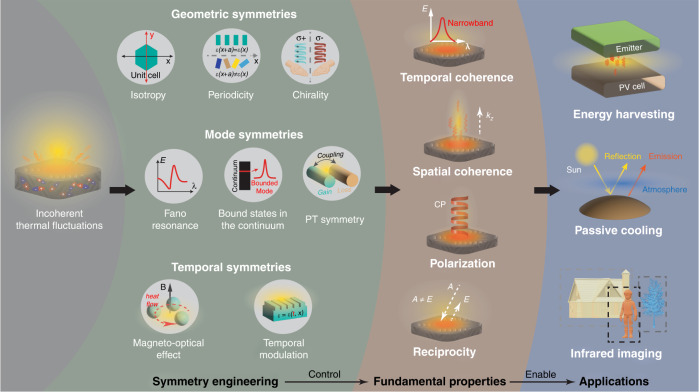
Symmetry Breaking in Thermal Photonics. Symmetry engineering provides a general framework to comprehensively tailor thefundamental properties of thermal radiation, advancing various thermal photonic applications

Lastly, the authors discuss magneto-optical effects which are intimately tied with temporal symmetries and reciprocity. The possibility to break Kirchhoff’s Law of thermal radiation opens up the door to approaching the fundamental limits of thermal energy conversion^8^. However, the requirement of strong magnetic fields to break reciprocity using magneto-optic media places severe technical challenges restricting its practical implementation. In this regard, the authors discuss spatiotemporal modulation as a promising alternative route to break reciprocity.

Many emerging topics in this field of symmetry engineering such as twist-optics and parity-time symmetry also have the potential to be adopted to thermal photonics effectively. The article from Liu et al. is very timely, and it is evident that the study of symmetries and symmetry breaking will be an exciting future area in thermal photonics.
